# Evaluating Anesthesia Guidance for Rescue Analgesia in Awake Patients Undergoing Carotid Endarterectomy with Cervical Plexus Blocks: Preliminary Findings from a Randomized Controlled Trial

**DOI:** 10.3390/jcm14010120

**Published:** 2024-12-28

**Authors:** Michał Jan Stasiowski, Nikola Zmarzły, Beniamin Oskar Grabarek

**Affiliations:** 1 Chair and Department of Emergency Medicine, Faculty of Medical Sciences in Zabrze, Medical University of Silesia, 40-555 Katowice, Poland; 2 Department of Anesthesiology and Intensive Care, 5th Regional Hospital, 41-200 Sosnowiec, Poland; 3 Collegium Medicum, WSB University, 41-300 Dabrowa Gornicza, Poland; nikola.zmarzly@gmail.com (N.Z.); bgrabarek7@gmail.com (B.O.G.)

**Keywords:** adequacy of anesthesia, carotid artery sheath infiltration, cervical plexus block, eversion carotid endarterectomy, surgical pleth index

## Abstract

**Background/Objectives:** Eversion carotid endarterectomy (CEA) in awake patients is performed using cervical plexus blocks (CPBs) with or without carotid artery sheath infiltration (CASI) under ultrasound guidance. Although adequacy of anesthesia (AoA) guidance monitors nociception/antinociception balance, its impact on intraoperative analgesia quality and perioperative outcomes in awake CEA remains unexplored. Existing literature lacks evidence on whether AoA-guided anesthesia enhances clinical outcomes over standard techniques. This study aimed to assess the role of AoA guidance in improving intraoperative analgesia and perioperative outcomes in patients undergoing CEA with CPBs alone or with CASI compared to standard practice. **Methods:** A randomized controlled trial included 184 patients divided into three groups: CPBs with intravenous rescue fentanyl (IRF) and lidocaine (LID) guided by hemodynamic observation (C group), AoA-guided IRF and LID (AoA group), and AoA-guided IRF, LID, and CASI (AoA-CASI group). Primary outcomes included perioperative adverse events, and secondary outcomes assessed rescue medication demand and hemodynamic stability. **Results:** Analysis of 172 patients revealed no significant differences between groups in perioperative adverse events or hemodynamic parameters (*p* > 0.05). However, the AoA-CASI group demonstrated significantly reduced IRF and LID usage compared to the C and AoA groups (*p* < 0.001). No significant advantage was observed between the AoA and C groups regarding adverse events (*p* = 0.1). **Conclusions:** AoA-guided anesthesia with or without CASI does not significantly reduce perioperative adverse events or improve hemodynamic stability in awake CEA. Clinical implications suggest that focusing on surgical technique optimization may yield greater benefits in reducing adverse events compared to advanced anesthetic monitoring. Further studies are warranted to explore alternative approaches to enhance clinical outcomes.

## 1. Introduction

Eversion carotid endarterectomy (CEA) is currently performed either under cervical plexus block (CPB) or general anesthesia (GA) [1,2]. Despite the technique used, stroke rates associated with the performance of eversion CEA remain at approximately 5% [3].

CPB is increasingly favored for facilitating eversion CEA in awake patients due to its ability to enable real-time neurological assessment during surgery [4]. However, failed blocks can result in significant intraoperative pain perception, known as nociception, which often necessitates rescue analgesia. Recent advances have explored the adjunctive use of preoperative carotid artery sheath infiltration (CASI) under ultrasound (US) guidance or intraoperative infiltrative anesthesia performed by surgeons to mitigate block failures [5,6]. Inadequate intraoperative analgesia, or insufficient antinociception, may lead to hemodynamic fluctuations, potentially contributing to the decomposition of atherosclerotic plaques and subsequent cardiocerebrovascular complications [5,7,8]. The concept of Adequacy of Anesthesia (AoA) monitoring addresses these challenges by providing a quantitative assessment of nociception/antinociception balance using tools such as the surgical pleth index (SPI) and entropy measures (response and state entropy, RE and SE). AoA monitoring has shown promise in enhancing the intraoperative efficacy of regional anesthesia in neurosurgery [9,10] and vitreoretinal surgery [11]. However, its application in vascular surgery, particularly in awake patients undergoing CEA, remains underexplored. A recent study highlighted the potential of AoA monitoring in reducing opioid use during regional anesthesia, suggesting its broader applicability in optimizing perioperative outcomes in high-risk populations.

The aim of this study was to determine the impact of AoA guidance in patients undergoing eversion CEA under CPB alone or with CASI on the hemodynamic stability and perioperative outcomes compared to standard practice.

## 2. Materials and Methods

### 2.1. Study Design

The study was carried out in accordance with the 1964 Declaration of Helsinki and was approved by the local bioethics committee at the Medical University of Silesia in Katowice (KNW/0022/KB1/102/15, 29 September 2015, Chairman M. Trusz-Gluza, Prof, MD). This study was also registered with the Clinical Trials Registry (SilesianMUKOAiIT4; NCT04500249; 1 October 2015), whereas the first patient was enrolled on 9 October 2015. Preoperatively, all patients were trained to use the numeric pain rating scale (NPRS) to determine pain perception and ensure homogeneity of the collected data.

In this prospective randomized study, a total of 184 patients with American Society of Anesthesiologists (ASA) scores of II-III were enrolled. Randomization was performed using a sealed-envelope method after each patient gave written informed consent to participate in the study and undergo preoperative peribulbar block for primary CEA.

The randomization sequence was generated using computer software to ensure unbiased allocation. This sequence was created by an independent statistician who was not involved in patient recruitment, intervention, or outcome assessment. A total of 184 opaque, sealed envelopes, each containing a group allocation (C group, AoA group, or AoA-CASI group), were prepared according to the randomized sequence. These envelopes were consecutively numbered and stored securely.

Once a patient consented to participate, the attending anesthesiologist opened the envelope corresponding to the patient’s sequential enrollment number. The envelope contained the group assignment pre-determined by the randomization list. The blinding process was implemented to minimize bias and ensure the integrity of the study’s outcomes. While the anesthesiologist administering the intervention could not be blinded due to the nature of the procedure, the surgical team and postoperative assessors remained blinded to group allocation. Postoperative assessments, including pain scores and adverse event documentation, were conducted by a separate team of clinicians who were not involved in the intraoperative management. This separation of roles ensured an unbiased evaluation of outcomes and maintained the reliability of the study results.

This step-by-step process demonstrates the rigor and transparency of the randomization method, ensuring reliability and minimizing bias in our study design.

The inclusion criteria were the following: age between 18 and 80 years, written informed consent to participate in the study, and surgical qualification to undergo CEA after Doppler ultrasonography of the intracranial arteries. The exclusion criteria were developed with specific rationales to ensure patient safety and maintain the reliability of the study outcomes. Patients with allergies to local anesthetics were excluded to prevent adverse reactions during regional anesthesia, a critical aspect of the procedure. Similarly, patients with significant neurological deficits from prior strokes were excluded to avoid confounding variables that could influence perioperative outcomes and obscure the effects of the intervention. Patients with impaired collateral circulation, as assessed through preoperative transcranial Doppler (TCD) testing, were excluded to minimize the risk of intraoperative neurological complications. These exclusions were designed to ensure a homogenous study population and reduce potential risks associated with the procedure.

The patients were divided into three groups: C group, classic technique of superficial/intermediate/deep block using Moore’s technique with ultrasound guidance performed at our center; AoA group, classic technique of superficial/intermediate/deep block using Moore’s technique with US and AoA monitoring of analgesia quality; and AoA-CASI combined technique group, classic technique of superficial/intermediate/deep block using Moore’s technique with US with AoA monitoring of analgesia quality combined with locoregional ultrasound-guided CASI.

### 2.2. Ultrasound Examination of Cerebral Circulation

Ultrasound imaging of the intracranial cerebral arteries was carried out by a neurologist with over 10 years of experience (D.K.; see Acknowledgements). Before the preoperative anesthesiologic visit and qualification for eversion CEA, each patient included in the study underwent ultrasound of the carotid and vertebral arteries and transcranial Doppler (TCD) examination to assess blood flow in the middle, anterior, and posterior cerebral arteries bilaterally and in the basilar artery [12] because it was proven to be a promising predictor of neurologic symptoms during ICA clamping [13]. This study aimed to select a homogeneous group of patients with a relatively low risk of perioperative neurological complications. The study included patients presenting with isolated internal carotid artery (ICA) stenosis without significant stenosis (>70%) of any of the other mentioned arteries. In addition, patients underwent an ICA compression test to assess the efficiency of collateral circulation through the anterior communicating artery on the opposite side of the operated common carotid artery (CCA) to evaluate transient hyperemic responses in the middle cerebral arteries after CCA compression (10 s) using TCD. Parameters describing autoregulatory function, such as return to baseline time, transient hyperemic response ratio, changes in vascular resistance, and interhemispheric blood flow (residual blood flow velocity), were compared. Preoperatively, all patients were trained to use the numeric pain rating scale (NPRS) to determine pain perception and ensure homogeneity of the collected data. The preoperative evaluation process adhered to the most recent European guidelines for patient selection and safety considerations, as outlined by Lamperti et al. [5], which emphasize standardized protocols to optimize patient outcomes and minimize perioperative risks. Patients were treated using intraarterial stents. The basilar, middle, anterior, and posterior cerebral arteries were examined according to accepted principles using a 2 MHz pulse-pencil transducer.

The signal from the basilar artery was obtained from a suboccipital approach at a depth of 70–90 mm, whereas the signals from the middle, anterior, and posterior cerebral arteries were obtained via an approach through the temporal bone scale at a depth of 50–60 mm, 65–75 mm, and 60–75 mm, respectively.

### 2.3. Eversion Carotid Endarterectomy

Standard monitoring procedures were performed throughout anesthesia and eversion CEA. Vital parameters included heart rate (HR), non-invasive blood pressure (NIBP), five lead electrocardiography (ECG), and arterial oxygen saturation (SaO_2_). The depth of anesthesia was monitored by entropy electroencephalogram (EEG) (SE and RE) to correlate SE values with possible loss of consciousness after carotid artery cross-clamping in all studied groups, and intraoperative analgesia was monitored using the surgical pleth index (SPI). The procedure was divided into four stages.

#### 2.3.1. STAGE 1

The EEG entropy sensor (RE, SE) was placed on the patient’s forehead contralateral to the operated site, the pulse oximeter (SPI) and the NIBP cuff on the finger on the side opposite to the surgical site, and a standard five-lead ECG on the patient’s back after admission to the operating room. Ten milliliters of the crystalloid solution per kilogram of body weight, according to each patient’s individual needs, was infused intravenously to rebalance overnight loss through respiratory and skin transpiration. Five liters of oxygen per minute was administered via a face mask or nasal cannula, according to each patient’s preference, to ensure perioperative comfort. The initial values were then recorded. Depending on group allocation, CPB was performed with or without CASI using a Stimuplex Ultra 22G/50 (B. Braun Melsungen AG, Melsungen, Germany) and an ultrasound system (M-Turbo; Sonosite, Bothell, Washington, DC, USA) with a 13 MHz linear probe (HFL38/13-6 MHz; Sonosite, Bothell, Washington, DC, USA).

All patients received a combined superficial/intermediate/deep cervical plexus block [14] using bupivacaine along with skin infiltration of the surgical incision site using 1% lidocaine (LID) [15] to block all potential nociceptive transmission from the operating field to differentiate the root cause of adverse events, either anesthesiologic underachievement or surgical manipulation.

Each patient was positioned supine with the head slightly elevated, and the head was turned away from the blocked side. The procedure began with an ultrasound scan of the lateral triangle of the neck. After skin disinfection, the ultrasound transducer was covered with a sterile cover and used with a sterile hypoallergenic transmission gel. The whole procedure of regional anesthesia was performed using a Stimuplex set (Stimuplex^®^ D 0.71 × 50 mm; B. Braun Melsungen AG, Melsungen, Germany) applying the in-plane technique.

##### SUPERFICIAL CERVICAL PLEXUS BLOCK

An ultrasound probe was placed at the middle of the anterior border of the sternocleidomastoid muscle. The needle was inserted at the C3 level, while the probe was maintained in a transverse position. Ten milliliters of 0.25% bupivacaine was injected to block the superficial branches after a negative blood aspiration test. Spread of the local anesthetic cranially and caudally along the lateral border of the sternocleidomastoid muscle was observed on ultrasonography.

##### INTERMEDIATE CERVICAL PLEXUS BLOCK

The needle tip was further advanced deeper under the sternocleidomastoid muscle and below the superficial fascia. A volume of 10 mL of 0.25% bupivacaine was injected after a negative blood aspiration test. The spread of local anesthetic was similarly observed on ultrasound.

##### DEEP CERVICAL PLEXUS BLOCK

The US ensured the proper direction of needle advancement towards the transverse process of the second, third, and fourth cervical vertebrae of the operated side until bone contact was achieved. After the needle was withdrawn at approximately 1 mm and correct needle placement was ensured, 3–5 mL of 0.5% bupivacaine (Bupivacainum Hydrochloricum 0.5% WZF; Polfa, Warsaw, Poland) was administered each time after negative blood aspiration, according to the patient’s anthropometry, to avoid overdosing of bupivacaine over 2 mg/kg/body weight [16]. Spread of local anesthetic was observed on the ultrasound monitor screen.

##### CASI

During surgery, local infiltration of the carotid sheath is often necessary because it is innervated by branches of the vagus nerve and the superior root of the ansa cervicalis, which are not anaesthetized by classic CPB [17]. Therefore, in the AoA-CASI group, after the superficial/intermediate/deep CPB, a technique similar to those used by Martusevicius et al. [18] was performed using six injections of 2 mL of 1% LID (Lignocainum Hydrochloricum 1% WZF; Polfa Warsaw SA, Poland) anteriorly and posteriorly to the CCA, ICA, and the external carotid artery (ECA), observing the circumfusion of local anesthetic on the ultrasound screen around the cervical arteries. In cases of poor visualization, posterior injections were not performed in the selected patients, and the LID volume was doubled anteriorly.

##### SKIN INFILTRATION

In all patients, subcutaneous infiltration was performed along the surgical incision line using 5 mL of 1% LID (Lignocainum Hydrochloricum 1% WZF; Polfa, Warsaw, Poland) along the medial border of the sternocleidomastoid muscle. The spread of the local anesthetic was observed on the ultrasound under the skin over the ICA and CCA.

The blocks were performed by the same anesthesiologist and intensive therapy specialist with over 10 years of experience in regional anesthesia (the first author). Sensory block tests were performed at 5 min intervals using wool swabs sprayed with ethanol and punctured with a 22G needle.

Eversion CEA was performed as described by Raithel [19] by a team of three vascular surgeons under the supervision of K. Sz. (see Acknowledgements), with over ten years of experience in this field and performing approximately 100 eversion CEAs per year.

#### 2.3.2. STAGE 2

The SPI values from 5 min after the detection of good-CPB, indicating ready to start eversion, to the beginning of sterilization of the surgical site, were considered to calculate mean SPI value. This allowed for calibration of the SPI sensor and measurement of preoperative parameters reflecting preoperative hemodynamic stability.

#### 2.3.3. STAGE 3 (INTRAOPERATIVELY)

In the AoA and AoA-CASI groups, the SPI scores were monitored *on-line* and recorded at 1 min intervals. If the ∆SPI value was 15 points above the ∆SPI value calculated in Stage 2, a rescue dose of fentanyl (FNT) at 1 µg/kg of body weight was administered intravenously (every 5 min) until the SPI value reached the ∆SPI value from Stage 2, and 5 mL of 1% LID (Lignocainum Hydrochloricum 1% WZF; Polfa, Warsaw, Poland) was administered to the operating site by the surgeon. In the C group, intraoperative rescue analgesia was administered according to each patient’s behavioral assessment, which was based on the observation of face mimics and grimaces, upper limb movements, vocalization of pain perception, change of hemodynamic parameters, cooperation with the surgical team, and was administered in response to surgical manipulations observed by an anesthesiologist who administered FNT similar to the AoA and AoA-CASI groups and stopped eversion CEA and administered additional rescue LID before further proceedings. Regardless of group allocation, if there was a risk of exceeding the cumulative dose of LID above 4.5 mg/kg body weight, no additional dose of local anesthetic was administered, and rescue analgesia was provided using only intravenous FNT. The duration of eversion CEA was defined as the period from the beginning of the skin incision to the placement of the last suture. We assumed that a rescue dose of FNT at 1 µg/kg body weight would provide sufficient rescue analgesia. In this study, ∆SPI > 15 was chosen as the threshold for nociception/antinociception intervention based on its established sensitivity in detecting insufficient analgesia while minimizing the risk of over-treatment [20]. Other thresholds, such as ∆SPI > 10 or absolute SPI > 50, were considered less appropriate for this context. The lower threshold of ∆SPI > 10 was associated with a higher rate of false positives, leading to excessive opioid administration, while absolute SPI > 50 was found to be less sensitive to incremental nociceptive changes, particularly during carotid endarterectomy. By adopting ∆SPI > 15, we aimed to balance the precision and safety of analgesia administration [21]. As an indicator for rescue analgesia in this study, as in our previous studies, we adopted a compromise protocol of ∆SPI value > 15 in comparison with the calculated Stage 2 baseline and lasting at least one minute. Our goal was to avoid FNT overdose resulting from the SPI value miscalculation due to its variability, possibly leading to apnea or severe bradycardia with hypotension. In cases of HR < 45, a rescue dose of 0.5 mg atropine (Atropinum sulfuricum; Polfa, Warsaw, Poland) was administered intravenously. When mean arterial pressure (MAP) was <60 mmHg, a rescue dose of ephedrine (Ephedrinum hydrochloricum; Polfa, Warsaw, Poland) was administered intravenously. In the case of MAP > 110 mmHg, a rescue dose of urapidil (Ebrantil 25; Takeda Pharma, Vienna, Austria) was administered intravenously when SPI values indicated proper antinociception in the AoA and AoA-CASI groups, or when anesthesiologic intuition led to the conclusion that hemodynamic instability was not triggered by insufficient antinociception.

#### 2.3.4. STAGE 4

In the postoperative care unit (PACU), all patients were further monitored (HR, SAP, MAP, DAP, SPI, SaO_2_) by the anesthesiology team that did not know which group the patients were assigned to. Each patient was monitored for 24 h for the presence of adverse effects, including vomiting (PONV), nausea, and allergic reactions, concurrently with a pain assessment. Ondansetron (Ondansetron Accord; Accord Healthcare Limited, Devon, UK) was administered intravenously at a single 4 mg dose when PONV occurred. When the MAP value was higher than 65 mmHg, a dose of 5 mL/kg body weight of optylite solution was infused. Oxygen was administered at a rate of 3 L/min via a face mask. Every 10 min, patients rated their pain intensity perception with the NPRS (0—no pain, 10—maximum pain). When an NPRS above 3 was recorded, according to guidelines for acute pain treatment, a standard dose of a nonsteroidal anti-inflammatory drug was administered [15,22]. As in the previous stage, SPI scores were monitored *on-line* and recorded at 1 min intervals. NPRS and SPI values were recorded for intolerable postoperative pain perception (IPPP, NPRS 4–10) and mild pain perception (NPRS 0–3). Patients were observed and monitored in the PACU for at least 30 min until they were transferred from the PACU. Before transferring patients to the Department of Vascular Surgery, they were monitored in the PACU for at least 30 min.

### 2.4. Statistical Analysis

The sample size was estimated at 159 using a one-way ANOVA based on a moderate effect size, an alpha level of 0.05, a power of 0.8, and division into three groups. G*Power 3.1.9.7 [23] was used for the calculations.

Statistical analysis was carried out using STATISTICA 13.3 (StatSoft, Kraków, Poland). The measured data are presented as mean and standard deviation (X ± SD) and median (Me) with interquartile range (IQR). Lack of normality of the data distribution was confirmed with the Shapiro–Wilk test. ANOVA, Kruskal–Wallis, and Dunn’s tests were then performed. Percentages are presented for nominal data. The relationships between nominal variables were verified using the chi-square test with Yates’ continuity correction. Statistical significance was set at *p* < 0.05.

## 3. Results

**Figure 1:**
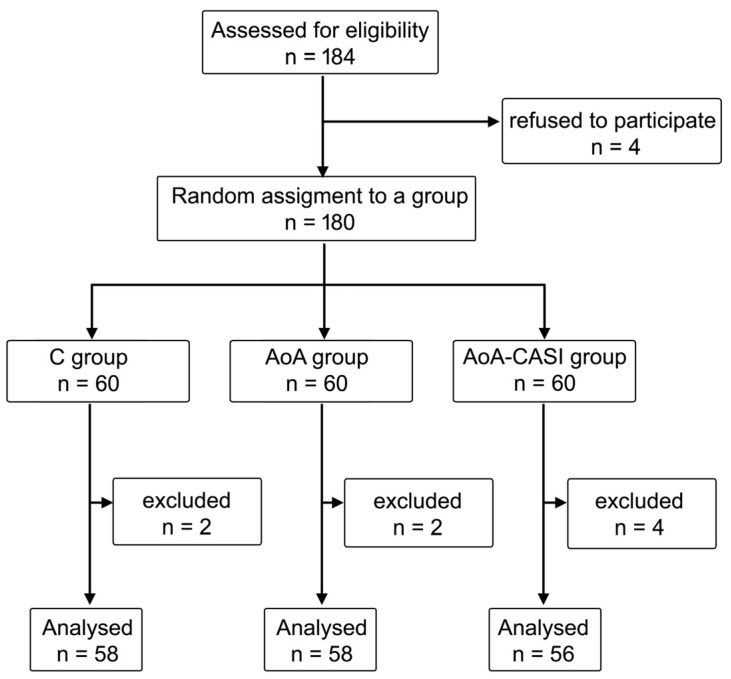
Randomization graph. C, classic technique; AoA, Adequacy of Anesthesia; CASI, carotid artery sheath infiltration.

A total of 184 patients participated in this study (Figure 1). Four patients refused to participate, and eight patients were excluded from the final analysis: four patients due to surgical reasons (loss of consciousness with significant drop in SE after CCA cross-clamping, three cases; intraoperative bleeding impairing hemodynamic stability, one case) and four patients due to anesthesiologic reasons (intraoperative technical problems with SPI monitoring as a result of patient arousal, one case; intraoperative heart arrhythmia, one case; technical problems with performance of CASI in the AoA-CASI group, two cases). Of the 172 patients, 56 (32.6%) were women, and 116 (67.4%) were men. The patients were divided into three groups: control (C) (33.7%), AoA (33.7%), and AoA-CASI (32.6%).

Detailed characteristics of the patients’ anthropometric data are shown in Table 1. No significant differences were observed between the individual groups in terms of age, height, weight, or Body Mass Index (BMI).

**Figure 2:**
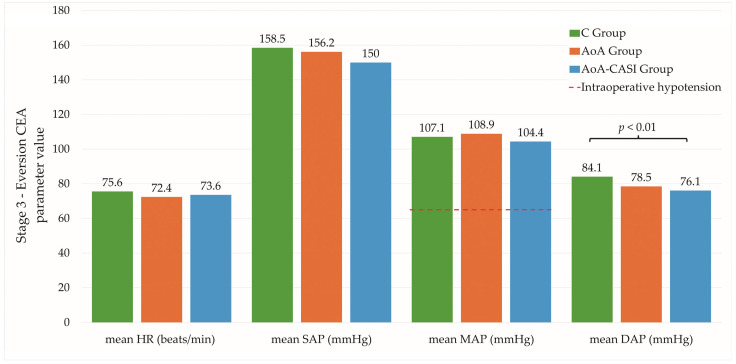
Hemodynamic stability during eversion CEA in the study groups. The dashed red line indicates a MAP of 65 mmHg, which is the threshold below which intraoperative hypotension occurs. C, classic technique; AoA, Adequacy of Anesthesia; CASI, carotid artery sheath infiltration; HR, heart rate; SAP, systolic blood pressure; MAP, mean arterial pressure; DAP, diastolic arterial pressure.

Significant differences in the mean SE values were observed in the first, second, and third stages (Table 2 and Table 3); however, these differences were not clinically relevant as they all fell within normal ranges. In addition, significant differences were reported for mean HR and MAP at stage 2. There were also differences between the C and AoA-CASI groups in terms of mean DAP at stage 3 (Figure 2). In the case of stage 4, only the differences between the C and AoA groups for minimum and maximum DAP were significant, although they fell within the normal range and were of no clinical value.

In the case of hemodynamic fluctuations, there were significant differences between the C and AoA-CASI groups in terms of maximum SAP and maximum DAP at stage 3 (Table 4). In addition, differences in maximum DAP were reported between the C and AoA-CASI groups. In stage 4, differences between the C and AoA groups were observed at the minimum and maximum DAP. However, they have little clinical relevance because they fall within the normal range.

**Figure 3:**
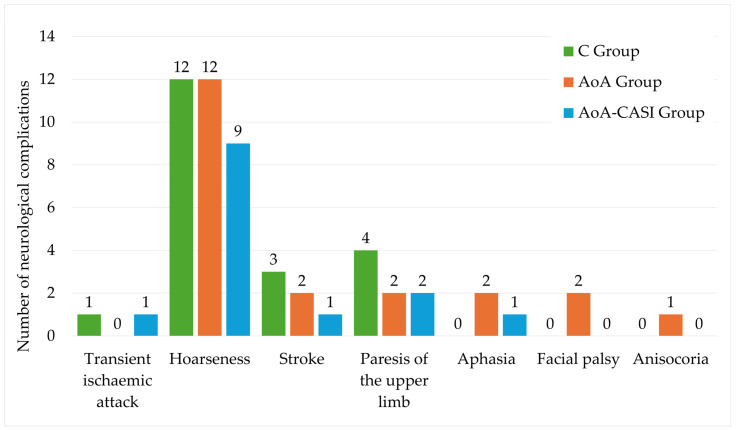
Incidence of neurological complications according to group allocation. C, classic technique; AoA, Adequacy of Anesthesia; CASI, carotid artery sheath infiltration.

The detailed complications in the studied groups are shown in Table 5 and Figure 3. No significant differences were observed between the studied groups.

**Figure 4:**
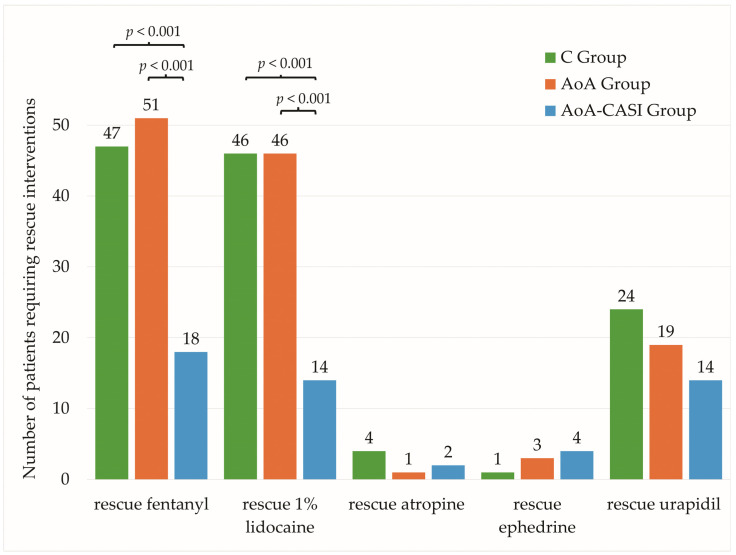
Number of patients requiring rescue interventions in the study groups. C, classic technique; AoA, Adequacy of Anesthesia; CASI, carotid artery sheath infiltration.

In the case of intraoperative interventions, significant relationships were observed between the number of patients requiring intravenous rescue fentanyl (IRF) and LID according to group allocation (Table 6, Figure 4).

**Figure 5:**
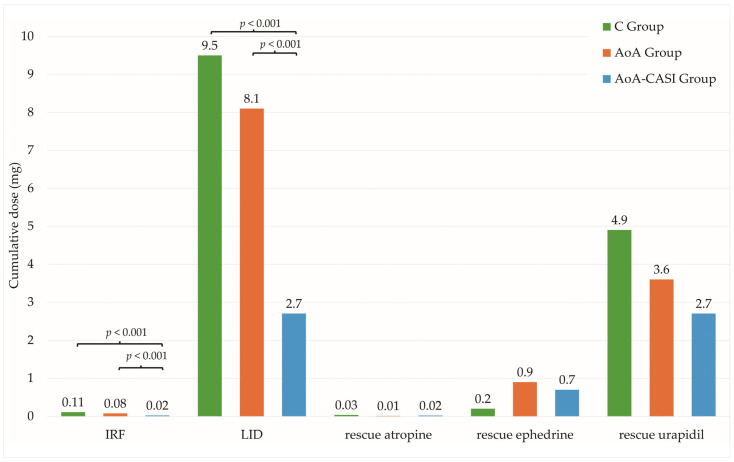
Cumulative dose of rescue interventions in the study groups. C, classic technique; AoA, Adequacy of Anesthesia; CASI, carotid artery sheath infiltration; IRF, intravenous rescue fentanyl; LID, lidocaine.

The relationship between the doses of rescue FNT and LID and group allocation was also significant (Table 6, Figure 5). The greatest differences were observed between the C and AoA groups. Although statistically significant differences were observed, they were not clinically significant because they did not translate into statistically significant differences for the rate of incidence of adverse events, which is the most important role in the recovery and quality of therapeutic value of performed surgical interventions.

## 4. Discussion

Unhealthy lifestyles and aging promote atherogenesis that may lead to significant ICA stenosis, which is reported in 5–7.5% of older adults [12], with the primary treatment being eversion CEA with or without patch angioplasty. Eversion CEA is usually performed under GA, either routinely with an intraoperative shunt [24,25], optionally to reduce the risk of new lesions in the brain [26], or as preferred by numerous operators using CPBs only in awake patients [6]. CPB in awake patients is especially important for those with commonly-observed cardiac risk [27] due to the ease of assessing neurological function during carotid artery cross-clamping, the stability of hemodynamic parameters, and the economic frugality associated mainly with length of hospital stay. Numerous anesthetic modalities of CPB are employed to facilitate the quality of perioperative outcomes, such as combinations of superficial/intermediate [28], deep block [29], and CASI [6]. To optimize perioperative outcomes, different additional techniques are added to CPBs in awake patients: infiltration of the incision line [30], mild sedation [31], adjuvants added to local anesthetics [32,33], the addition of a mandibular nerve block [34], or even music [35].

Despite the use of regional anesthesia techniques, antinociception may fail fully or partially due to insufficient block for numerous reasons [36]. In this study, this was observed in seven cases (4.07%), which was reflected in the incidence rate of IPPP. Insufficient antinociception was detected by observing hemodynamic parameters under GA or in cooperation with patients under regional anesthesia. Currently, several digital techniques for nociception/antinociception balance monitoring have been introduced into everyday practice, such as the surgical pleth index, antinociception index, pupillometry, and nociception level [37].

In the AoA-CASI group, we analyzed a multi-model treatment plan, including ultrasound-guided deep/intermediate/superficial block with CASI and AoA-guided rescue FNT and 1% LID administration in the case of a partially failed block. In the current literature, CASI is controversial, as it has been observed to complicate the procedure and provide no benefits in terms of hemodynamic stability and reduced rescue intraoperative analgesia [38]. Contrary to the abovementioned study, we observed a significant reduction in the demand for rescue analgesia using both FNT and LID between the AoA-CASI group and the other two groups, with no advantage in the incidence of perioperative adverse events. Tasar et al. observed cerebrovascular accidents in 7 of 126 patients (5.5%) [27], and performed eversion CEA without supplemental topical LID in 23% and 7% of patients in the intermediate and superficial block groups, respectively [39], which is a slightly higher rate than that in the current study.

For rescue analgesia using LID in patients undergoing eversion CEA under CPB, ultrasound-guided intermediate cervical block used either 20 mL of 0.75% ropivacaine or 20 mL of 0.375% ropivacaine with 20 mL of 1% prilocaine. The blocks were carried out by ultrasound-guided infiltration below the sternocleidomastoid muscle. In the study by Koköfer et al., the carotid sheath was infiltrated under US, and supplementary LID was given: 5.0 (±3.63) ml in the 0.375% ropivacaine group and 5.17 (±2.76) ml in the 0.75% ropivacaine group (*p* = 0.846) [40]. They achieved slightly better results compared to the current study findings, as ropivacaine provides a higher quality sensory block compared to the bupivacaine used in our center at the time of the study.

In the current study, no cases of acute respiratory failure requiring upper airway management were observed, whereas stroke was observed in 6 of 172 cases (3.48%), with no significant differences between the groups.

In the current study, we investigated the utility of AoA monitoring in patients undergoing eversion CEA under CPB with or without CASI and compared it with standard practice. Despite the group allocation, out of 172 analyzed cases, we observed the need for rescue intravenous analgesia in 116 cases (67.4%) that required an average cumulative FNT dose of 69.9 ± 64.7 µg, and rescue intraoperative analgesia in 106 cases (61.6%) that required an average cumulative dose of 6.8 ± 6.9 mL of 1% LID. In 10 patients, no further dose of rescue LID was administered because of the risk of exceeding the cumulative dose, possibly resulting in systemic complications [41].

When comparing the control group with the AoA and AoA-CASI groups, we observed a reduced number of patients requiring rescue intravenous FNT (47 [81%] vs. 51 [87.9%] vs. 18 [32.1%], respectively; *p* < 0.001), reduced average requirement for cumulative FNT dose (109.5 ± 69.7 µg vs. 76.2 ± 51 µg vs. 22.3 ± 35.6 µg, respectively; *p* < 0.001), reduced number of patients requiring rescue intraoperative LID (46 [79.3%] vs. 46 [79.3%] vs. 14 [25%], respectively; *p* < 0.001), and a reduced average requirement for cumulative LID dose (95 ± 70 mg vs. 81 ± 64 mg vs. 27 ± 55 mg, respectively; *p* < 0.001).

However, the abovementioned reduction did not result in improved hemodynamic stability or lead to a reduction in perioperative neurological adverse events. Chamseddine H et al. had a similar observation and reported no benefit of performance of CASI over GA in patients undergoing CEA [42]. In the case of the number of patients requiring rescue administration of vasoactive drugs, such as atropine, ephedrine, and urapidil, no significant differences were observed between the groups in terms of the number of patients and demand for the average dose, which may indirectly reflect the lack of statistically and clinically significant differences in terms of hemodynamic stability between the groups.

The addition of CASI to the AoA guidance of CPB did not result in a reduction of adverse events compared to the control and AoA groups. However, previous results have shown that peribulbar blocks added as preventive analgesia techniques to AoA-guided GA did not reduce the incidence rate of IPPP and hemodynamic instability in patients undergoing vitreoretinal surgeries [11].

Fluctuations in hemodynamic parameters may lead to intraoperative decompensation of atherosclerotic plaques [43], with ensuing cardiovascular consequences [44]. Intraoperative hypotension is a major concern for the anesthesiologist because even short durations of low MAP have been associated with adverse postoperative clinical outcomes [45]. However, intraoperative hypertension, most frequently observed among patients with older ages, a BMI of 25, and hypertension as a coexisting disease were proven to likely result in hypertension during the postoperative period in a significant number of patients [46]. Both intraoperative hypotension and hypertension may result from excessive or suboptimal nociception or antinociception, although permissive hypertension before declamping of CCA has been found to be beneficial due to recruitment of the cerebral collateral network, and it substantially reduces the rate of adverse events and need for shunting in awake/sedated patients [47].

The AoA concept involved assessing the depth of GA using a forehead sensor to measure the EEG parameters (RE and SE) and the SPI derived from finger photoplethysmography [48]. In patients undergoing surgical procedures, entropy EEG is monitored to achieve proper depth of the hypnotic component of GA, either using intravenous or volatile anesthetics. Adequate suppression of the limbic system for GA is obtained within a range of 45–60 SE [49] or within a range of 60–80 during deep sedation [50]. In the current study, SE was monitored only to detect early brain function lesions. However, SPI guidance for intravenous FNT administration during TIVA, based on the formula where SPI = 100 − (0.67 × PPGAnorm + 0.33 × HBInorm), collected from finger photoplethysmography, has been previously shown to not require complex and time-consuming preparations [51]. It is also more effective comparing to the guidance based on the hemodynamic parameters observation in response to intraoperative vasoactive reactions after surgery [52]. Fluctuations in SPI values in response to nociceptive stimulation have been shown to correlate with serum opioid concentration [53]. The SPI value increase at certain noxious stages of specific surgical manipulations (0—no painful stimulation, 100—maximal painful stimulation) after the occurrence of painful stimulus and further return to the baseline level after a bolus (antinociception) of IRF (1 µg/kg body weight and 5 mL of 1% LID) eases AoA guidance [54], as demonstrated in our previous study [55]. The SPI has been effectively utilized to monitor intraoperative and postoperative analgesia and also assist in guiding IRF [56]. Employment of SPI guidance for IRF administration has been shown to lead to fewer adverse events, decreased opioid consumption, quicker emergence from GA [57,58], and reduced postoperative pain [59], as compared to IRF administration based on the observance of hemodynamic fluctuations and anesthesiologic intuition. The observation of SPI value fluctuations at different stages of surgery has also been shown to predict IPPP in individual cases [60,61,62,63]; countermeasures are therefore taken for prevention before emergence from GA, for example, by administration of extra doses of medication with antinociceptive mechanisms of action.

As SE and RE of patients have been proven to be useful in assessing the depth of anesthesia, comparable conditions have been created between groups and individual patients in terms of the perception of intraoperative nociception using SPI, which is used to determine the response of the central nervous system to nociception during surgery [64,65].

Regional anesthesia techniques, such as pectoral nerve block type II for breast surgery [10], abdominal wall blocks for single-port laparoscopic hernia surgery [9], and infiltration anesthesia for spine surgery [7], have been shown to reduce opioid consumption and pain scores using SPI guidance. Similarly, in the current study, the performance of CASI, serving as an additional block of the vagal nerve branches and the superior root of the ansa cervicalis, which are not anesthetized with classical CPB (see Methodology section), resulted in a reduced demand for rescue FNT and LID compared with that in standard practice and that observed in the AoA group.

In the current study, we investigated the impact of AoA guidance for IRF and LID administration in patients receiving CPB alone or with CASI in the case of suboptimal antinociception on the rate of incidence of adverse events. We observed that AoA guidance, regardless of whether additional CASI was applied, was superior to the standard practice of administering IRF and LID based on the observation of hemodynamic parameters and patient cooperation. This is because an increase in SPI value appeared faster than standard parameters and the patients’ experience of insufficient nociception. However, the administration of rescue analgesia and other medications influencing hemodynamic parameters resulted in comparable hemodynamic parameter values, ensuring patient safety despite group allocation. We did not observe superiority of AoA guidance over standard practice in terms of patient safety. As MAP < 60 mmHg is associated with myocardial infarction, acute kidney injury, and death as a function of not only severity but also duration [66], the observed differences in hemodynamic parameters in the current study were not of clinical value (see Table 2, Table 3 and Table 4). Presumably, a statistically significant reduction in the demand for rescue analgesia using FNT and LID contributed to improved patient and operator satisfaction, as the incidence of insufficient antinociception was lower under AoA guidance, which will be analyzed in detail separately at each stage of eversion CEA. Questionnaires were not used to validate patient or surgeon satisfaction.

The efficacy of CPB is dependent on the carotid artery bifurcation level, and it influences the incidence of adverse neurological events [67], which were not analyzed in the current study because such an observation was published after randomization was completed. We also intentionally did not study a group that underwent CPB with CASI without AoA guidance because such studies are present and their results are known; therefore, we mainly focused on the effectiveness of AoA guidance in CPB with or without CASI as a potential new direction compared to the classic CPB technique most widely used in our center. Similarly, in our previous studies concerning the utility of AoA monitoring on perioperative outcomes [7,50,68], the following will be presented separately: relationships between intraoperative nociception detection using AoA indices at each stage of operation and hemodynamic stability [55], the incidence of postoperative nausea and vomiting [69,70], relationships between hemodynamic stability and AoA indices at each stage of eversion CEA, relationships between dose of rescue analgesia intraoperatively and PONV, relationships between SPI values and postoperative pain perception, and relationships between SE fluctuations and incidence of neurological dysfunctions, especially during loss of consciousness following CCA cross-clamping or declamping. Finally, AoA monitoring in patients undergoing eversion CEA is challenging because most have general atherosclerosis, which proportionately affects peripheral blood flow and may therefore proportionately impair the quality of the collected data.

### Strengths and Limitations of the Study

The strengths of this study lie in its rigorous randomized controlled trial design, which is widely regarded as the gold standard for clinical research and ensures unbiased participant allocation and robust internal validity. The inclusion of a well-defined patient population with American Society of Anesthesiologists (ASA) scores of II–III, along with comprehensive and explicitly rationalized exclusion criteria, contributed to the reliability and generalizability of the findings by minimizing potential confounding variables. The study also utilized advanced monitoring techniques, such as Adequacy of Anesthesia (AoA) guidance and surgical pleth index (SPI) monitoring, providing a novel approach to assessing nociception/antinociception balance in awake patients undergoing CEA in order to investigate its impact on rate of incidence of adverse events. Detailed and transparent reporting of both surgical and anesthetic procedures, alongside meticulous documentation of complications and adverse events, further enhances the reproducibility and clinical applicability of the results. However, the study is not without limitations. Its single-center nature may limit the generalizability of the findings to other healthcare settings with differing patient populations and clinical practices. Additionally, the lack of long-term follow-up data restricts the ability to evaluate the enduring impact of AoA monitoring on postoperative outcomes. Variability in operator proficiency and patient-specific anatomical factors, inherent to regional anesthesia, may also influence the reproducibility of the techniques described. Despite these limitations, the study provides valuable insights into the optimization of perioperative care for patients undergoing CEA.

## 5. Conclusions

AoA guidance for CPB with or without CASI reduced the need for intraoperative rescue analgesia but neither reduced perioperative adverse events nor improved hemodynamic stability, as compared to the two other groups. While AoA monitoring enhances analgesia administration efficiency, further studies are needed to optimize surgical and anesthetic strategies for better patient outcomes. These findings underline the importance of tailoring anesthetic approaches to individual patient needs.

## Figures and Tables

**Table 1:** Anthropometric data of patients enrolled in this study.

Anthropometric Data		Total	C Group	AoA Group	Aoa-CASI Group	*p*-Value
		n = 172 (100%)	n = 58 (33.7%)	n = 58 (33.7%)	n = 56 (32.6%)	
Age X ± Sd Me (IQR)	years	69.3 ± 7.1 69 (9)	69.4 ± 7.8 69.5 (11)	69.4 ± 7.9 70 (12)	69 ± 5.4 69 (7)	0.90 NS
Height X ± Sd Me (IQR)	cm	167.1 ± 9.3 168 (11)	167.1 ± 7.9 168 (12)	167.4 ± 7.4 168 (8)	166.8 ± 12.1 168 (14)	1.0 NS
Weight X ± Sd Me (IQR)	kg	76.2 ± 13.2 75.5 (20)	75 ± 14.2 77 (22)	77.8 ± 13.2 77 (16)	75.8 ± 12.2 75 (18.5)	0.69 NS
BMI X ± Sd Me (IQR)	kg/m^2^	27.4 ± 4.9 274 (5.9)	26.8 ± 4.2 27.7 (6.9)	27.8 ± 4.7 27.9 (6.2)	27.5 ± 5.9 27 (5.1)	0.72 NS
BMI N (%)	underweight	2 (1.2%)	2 (3.4%)	0 (0%)	0 (0%)	0.60 NS
	normal	53 (30.8%)	17 (29.3%)	19 (32.8%)	17 (30.4%)	
	overweight	73 (42.4%)	24 (41.4%)	23 (39.7%)	26 (46.4%)	
	obese	44 (25.6%)	15 (25.9%)	16 (27.6%)	13 (23.2%)	
Sex N (%)	male	116 (67.4%)	42 (72.4%)	37 (63.8%)	37 (66%)	0.59 NS
	female	56 (32.6%)	16 (27.6%)	21 (36.2%)	19 (33.9%)	

C, classic technique; AoA, Adequacy of Anesthesia; CASI, carotid artery sheath infiltration; Sd, standard deviation; Me, median; IQR, interquartile range; kg, kilogram; BMI, body mass index; NS, not significant.

**Table 2:** Parameter values measured in Stage 1 in studied groups.

Parameter	C Group	AoA Group	AoA-CASI Group	*p*-Value
	n = 58 (33.7%)	n = 58 (33.7%)	n = 56 (32.6%)	
Stage 1—Onset				
HR (beats/min)	68.6 ± 11.3 68 (17)	68.5 ± 10 66.5 (12)	71.4 ± 13.2 69 (18)	0.58 NS
SAP (mmHg)	154.6 ± 24.6 158 (33)	151.1 ± 27.4 151.5 (32)	152.5 ± 22.5 154 (37)	0.73 NS
MAP (mmHg)	106.1 ± 20.2 105.5 (28)	107.3 ± 15.3 108.5 (17)	106.9 ± 14.5 105 (23)	0.69 NS
DAP (mmHg)	76.6 ± 10.8 75.5 (13)	75 ± 11.6 74 (16)	76.4 ± 12.3 76 (13)	0.76 NS
SE	87.7 ± 2.5 89 (2)	88.4 ± 2.4 89 (2)	87.7 ± 2 88 (2)	AoA vs. AoA-CASI, *p* = 0.03
SPI	-	57 ± 15.2 57 (18)	59.6 ± 16.8 64 (26)	1.0 NS

C, classic technique; AoA, Adequacy of Anesthesia; CASI, carotid artery sheath infiltration; HR, heart rate; SAP, systolic blood pressure; MAP, mean arterial pressure; DAP, diastolic arterial pressure; SE, state entropy; SPI, surgical pleth index.

**Table 3:** Mean values of measured parameters in Stages 2, 3, and 4 in studied groups.

Parameter	C Group	AoA Group	AoA-CASI Group	*p*-Value
	n = 58 (33.7%)	n = 58 (33.7%)	n = 56 (32.6%)	
Stage 2—After CPB				
mean HR (beats/min)	70.3 ± 14 67 (20)	68.9 ± 12 67.5 (17)	76.6 ± 14.1 74 (20)	C vs. AoA-CASI, *p* = 0.03 AoA vs. AoA-CASI, *p* = 0.01
mean SAP (mmHg)	148.3 ± 28.3 148.5 (36)	155.5 ± 26.4 156 (45)	157.6 ± 26.9 160 (32)	0.8 NS
mean MAP (mmHg)	101 ± 19.1 100 (28)	107.6 ± 16 108.5 (19)	112.8 ± 18.5 113 (20)	C vs. AoA-CASI, *p* = 0.003
mean DAP (mmHg)	77.4 ± 14.4 75.5 (14)	77 ± 14.1 77 (18)	80.3 ± 13.7 81 (19)	0.30 NS
mean SE	85.9 ± 6.1 88 (4)	84.1 ± 9.5 88 (3)	80.2 ± 12.9 50 (49)	C vs. AoA-CASI, *p* < 0.001 AoA vs. AoA-CASI, *p* < 0.001
mean SPI	-	53.3 ± 23.5 47.3 (42.7)	79.3 ± 15.8 87 (14)	1.0 NS
Stage 3—Eversion CEA				
mean HR (beats/min)	75.6 ± 13.4 74.5 (19)	72.4 ± 12.1 71.1 (18.9)	73.6 ± 13.3 71.7 (17.1)	0.48 NS
mean SAP (mmHg)	158.5 ± 23.3 160.1 (35.4)	156.2 ± 21.1 157 (27.6)	150 ± 23.7 155 (29.5)	0.13 NS
mean MAP (mmHg)	107.1 ± 15.1 107.1 (22.5)	108.9 ± 13 110.7 (16.1)	104.4 ± 14.6 104.6 (20)	0.35 NS
mean DAP (mmHg)	84.1 ± 12.8 82.9 (18.3)	78.5 ± 12 77.3 (16)	76.1 ± 13.6 76.8 (22.6)	C vs. AoA-CASI, *p* < 0.01
mean SE	87.1 ± 2.5 87.4 (3.3)	86.9 ± 4.2 88 (2)	88 ± 2.5 88.4 (1.8)	C vs. AoA-CASI, *p* < 0.02
mean SPI	-	42.2 ± 10.8 41.4 (14.5)	44.3 ± 10.8 44.5 (17.6)	1.0 NS
Stage 4—PACU				
mean HR (beats/min)	77.2 ± 13.7 77 (15.8)	71.9 ± 12.3 71.3 (17.7)	73.2 ± 13.4 72.6 (17.2)	0.11 NS
mean SAP (mmHg)	156.7 ± 25.5 158 (29)	153 ± 25.4 154 (22.8)	159.3 ± 21 162.2 (31.3)	0.45 NS
mean MAP (mmHg)	105.7 ± 15.7 107.5 (20.5	106.4 ± 13.5 109 (15.8)	110 ± 13.7 112.3 (17.7)	0.22 NS
mean DAP (mmHg)	83.3 ± 12.8 84 (16)	75.9 ± 12.5 75 (15.7)	79.5 ± 12.4 79 (19.7)	C vs. AoA, *p* = 0.004
mean SPI	-	45.2 ± 15.4 43.1 (21.9)	50.6 ± 14.8 52.3 (23.2)	1.0 NS

C, classic technique; AoA, Adequacy of Anesthesia; CASI, carotid artery sheath infiltration; CPB, cervical plexus block; CEA, carotid endarterectomy; PACU, postoperative care unit; HR, heart rate; SAP, systolic blood pressure; MAP, mean arterial pressure; DAP, diastolic arterial pressure; SE, state entropy; SPI, surgical pleth index.

**Table 4:** Comparison of hemodynamic fluctuations in studied groups.

Parameter	C Group	AoA Group	AoA-CASI Group	*p*-Value
	n = 58 (33.7%)	n = 58 (33.7%)	n = 56 (32.6%)	
Stage 3—Eversion CEA				
max HR (beats/min)	87.6 ± 18.4 85.5 (27)	83.8 ± 15.4 83.5 (17)	85.2 ± 14.6 85 (19)	0.66 NS
max SAP (mmHg)	189.4 ± 34 189 (36)	184 ± 25.5 187 (35)	173.9 ± 26.8 178 (32)	C vs. AoA-CASI, *p* < 0.03
max MAP (mmHg)	128 ± 19.7 127 (27)	125.2 ± 15.7 125 (20)	119.4 ± 16.3 120 (22)	0.05 NS
max DAP (mmHg)	98.5 ± 20.4 96.5 (28)	89.5 ± 12.1 88.5 (16)	87.1 ± 14.4 89 (21)	C vs. AoA, *p* = 0.007 C vs. AoA-CASI, *p* = 0.002
max SE	89.9 ± 1.7 90 (2)	90.4 ± 0.8 91 (1)	90.1 ± 2.6 91 (1)	0.13 NS
max SPI	-	72.5 ± 15.4 76 (21)	72.3 ± 14.1 75 (21)	1.0 NS
min HR (beats/min)	63.8 ± 11.9 64 (18)	61.9 ± 12.2 61.5 (16)	64.1 ± 16.4 61 (18)	0.56 NS
min SAP (mmHg)	130.3 ± 31 130.5 (34)	128.8 ± 26 130.5 (37)	125.1 ± 30.1 132 (48)	0.91 NS
min MAP (mmHg)	85.2 ± 17.9 85 (24)	91.4 ± 18.5 94 (23)	89.1 ± 20 92 (32)	0.21 NS
min DAP (mmHg)	68 ± 12.3 67 (14)	64.9 ± 13.8 65 (20)	65.6 ± 16.5 66 (28)	0.5 NS
min SE	79.6 ± 11.8 84 (5)	79.9 ± 13.8 85 (5)	80.3 ± 15.2 85 (6)	0.1 NS
min SPI	-	19.4 ± 8.7 17 (13)	22.5 ± 9.2 21 (10)	1.0 NS
Stage 4—PACU				
max HR (beats/min)	80.3 ± 14.3 80 (19)	77.1 ± 14.4 76 (18)	77.7 ± 13.8 77 (22)	0.37 NS
max SAP (mmHg)	163.9 ± 25.6 166 (38)	163.6 ± 23.9 166 (24)	169.2 ± 22.6 175 (32.5)	0.35 NS
max MAP (mmHg)	111.3 ± 18.5 111.5 (27)	112.2 ± 14.1 114 (14)	116.3 ± 14.5 118.5 (17.5)	0.19 NS
max DAP (mmHg)	88.6 ± 15.9 89 (22)	80.6 ± 13 82 (17)	84.6 ± 14.6 86 (24.5)	C vs. AoA, *p* = 0.02
max SPI	-	62.3 ± 17.9 59 (29)	64.2 ± 14.5 66 (20)	1.0 NS
min HR (beats/min)	73.7 ± 13.7	68.1 ± 11.7 67 (15)	70.4 ± 13.3 69 (19)	0.08 NS
min SAP (mmHg)	150 ± 26.1 150 (32)	144.7 ± 24.9 147 (30)	149.9 ± 22.1 153.5 (32)	0.41 NS
min MAP (mmHg)	101.6 ± 13.7 104 (22)	100.6 ± 14.8 103 (17)	104. ± 14.7 106 (21)	0.39 NS
min DAP (mmHg)	79.6 ± 14 77 (19)	71.1 ± 13.5 69 (18)	75.3 ± 11.7 75.5 (19.5)	C vs. AoA, *p* = 0.002
min SPI	-	31.6 ± 16 31 (20.5)	36.5 ± 16.3 31 (27.5)	1.0 NS

C, classic technique; AoA, Adequacy of Anesthesia; CASI, carotid artery sheath infiltration; CEA, eversion carotid endarterectomy; PACU, postoperative care unit; max, maximum; min, minimum; HR, heart rate; SAP, systolic blood pressure; MAP, mean arterial pressure; DAP, diastolic arterial pressure; SE, state entropy; SPI, surgical pleth index.

**Table 5:** Complications in the studied groups.

Complications	Total	C Group	AoA Group	AoA-CASI Group	*p*-Value
	Postoperative pain perception				
NPRS	0.4 ± 1.1 0 (0)	0.4 ± 1.2 0 (0)	0.3 ± 0.9 0 (0)	0.4 ± 1.3 0 (0)	0.99 NS
Number of patients with IPPP (NPRS 4–10)	7 (4.07%)	3 (5.17%)	1 (1.72%)	3 (5.36%)	0.5 NS
	Neurological complications (% of incidence)				
Transient ischemic attack	2 (1.16%)	1 (1.72%)	0 (0%)	1 (1.79%)	1.0 NS
Hoarseness	33 (19.19%)	12 (20.69%)	12 (20.69%)	9 (16.07%)	0.8 NS
Stroke	6 (3.49%)	3 (5.17%)	2 (3.45%)	1 (1.79%)	0.6 NS
Paresis of the upper limb	8 (4.65%)	4 (6.9%)	2 (3.45%)	2 (3.57%)	0.6 NS
Aphasia	3 (1.74%)	0 (0%)	2 3.45%)	1 (1.79%)	0.4 NS
Facial palsy	2 (1.16%)	0 (0%)	2 (3.45%)	0 (0%)	0.1 NS
Anisocoria	1 (0.58%)	0 (0%)	1 (1.72%)	0 (0%)	0.4 NS

C, classic technique; AoA, Adequacy of Anesthesia; CASI, carotid artery sheath infiltration; IPPP, intolerable postoperative pain perception; NPRS, numerical pain rating score; NS, not significant.

**Table 6:** Number of patients requiring intraoperative interventions.

Intraoperative Rescue Intervention	Total	C Group	AoA Group	AoA-CASI Group	*p*-Value
	n = 172 (100%)	N = 58 (33.7%)	n = 58 (33.7%)	N = 56 (32.6%)	
Number of patients requiring IRF	116 (67.4%)	47 (81%)	51 (87.9%)	18 (32.1%)	C vs. AoA-CASI, *p* < 0.001 AoA vs. AoA-CASI, *p* < 0.001 C vs. AoA, *p* = 0.4
Cumulative dose of requiring IRF (µg)	69.9 ± 64.7 50 (100)	109.5 ± 69.7 100 (100)	76.2 ± 51 50 (50)	22.3 ± 35.6 0 (50)	C vs. AoA-CASI, *p* < 0.001 AoA vs. AoA-CASI, *p* < 0.001 C vs. AoA, *p*=0.1
Number of patients requiring rescue LID	106 (61.6%)	46 (79.3%)	46 (79.3%)	14 (25%)	C vs. AoA-CASI, *p* < 0.001 AoA vs. AoA-CASI, *p* < 0.001 C vs. AoA, *p* = 1.0
Cumulative dose of rescue 1% LID (mg)	6.8 ± 6.9 5 (10)	9.5 ± 7 10 (10)	8.1 ± 6.4 9 (5)	2.7 ± 5.5 0 (2.5)	C vs. AoA-CASI, *p* < 0.001 AoA vs. AoA-CASI, *p* < 0.001 C vs. AoA, *p* = 1.0
Number of patients requiring rescue atropine	7 (4.1%)	4 (6.9%)	1 (1.7%)	2 (3.6%)	0.4 NS
Cumulative dose of rescue atropine (µg)	20.5 ± 113.2 0 (0)	34.5 ± 158.4 0 (0)	8.6 ± 65.7 0 (0)	18.2 ± 94.5 0 (0)	0.4 NS
Number of patients requiring rescue ephedrine	8 (4.7%)	1 (1.7%)	3 (5.2%)	4 (7.3%)	0.4 NS
Cumulative dose of rescue ephedrine (mg)	0.6 ± 3 0 (0)	0.2 ± 1.3 0 (0)	0.9 ± 4.3 0 (0)	0.7 ± 2.6 0 (0)	0.4 NS
Number of patients requiring rescue urapidil	57 (33.3%)	24 (41.4%)	19 (32.8%)	14 (25.5%)	0.2 NS
Cumulative dose of rescue urapidil (mg)	3.8 ± 6.7 0 (5)	4.9 ± 7.6 0 (10)	3.6 ± 6.6 0 (5)	2.7 ± 5.8 0 (5)	0.2 NS

C, classic technique; AoA, Adequacy of Anesthesia; CASI, carotid artery sheath infiltration; IRF, intravenous rescue fentanyl; LID, lidocaine; mg, milligram; µg, microgram; NS, not significant.

## Data Availability

The original contributions inquiries can be directed to the corresponding author.
